# The Relationship between Sleep Quality and Social Intimacy, and Academic Burn-Out in Students of Medical Sciences

**DOI:** 10.5539/gjhs.v8n5p231

**Published:** 2015-11-05

**Authors:** Azizollah Arbabisarjou, Hashemi Seyed Mehdi, Mohammad Reza Sharif, Kobra Haji Alizadeh, Peyman Yarmohammadzadeh, Zahra Feyzollahi

**Affiliations:** 1 Pregnancy Health Research Center, Zahedan University of Medical Sciences, Zahedan, Iran; 2 Department of Pediatrics, Kashan University of Medical Sciences, Kashan, Iran; 3 Department of Psychology Bandar Abbas Branch, Islamic Azad University, Bandar Abbas, Iran; 4 Azarbaijan Shahid Madani University, Tabriz, Iran

**Keywords:** sleep quality, social intimacy, academic burn-out, medical students

## Abstract

**Introduction:**

Academic burnout leads to creation of a series of negative and scattered thoughts, loss of hope and emotional and physical exhaustion in carrying out activities. Two factors that affect academic burnout are sleep quality and social intimacy. This study was conducted in order to investigate the relationship between sleep quality and social intimacy, and academic burn-out in the students of Tabriz University of Medical Sciences

**Materials & Methods:**

This study was descriptive and correlational. The population of this study consisted of the students in Tabriz University of Medical Sciences and 196 medical students were selected. They completed Berso et al. Academic Burnout Questionnaire, Pittsburgh Sleep Quality Index (PSQI) and Miller Social Intimacy Scale (MSIS). The validity of the questionnaires confirmed by experts’ views. Their reliability were obtained as 77%, 64% and 85% for academic burnout, sleep quality and social intimacy questionnaires respectively by calculating the internal consistency (Cronbach’s alpha). For data analysis, descriptive statistics and Pearson correlation test, Regression, cluster analysis and t-test were used.

**Results:**

The results showed that there was a positive and significant relationship between sleep quality and academic burnout at the level p<0.05 (r=0.38). There was a negative and significant relationship between social intimacy and academic burnout at the level p<0.05 (r=-0.40). Also, the regression results showed that sleep quality and social intimacy were able to predict 37% and 39% of academic burnout respectively. Moreover, the students were divided into two clusters of individuals with high social intimacy and individuals with low social intimacy. No significant difference was found between the two types in terms of the variable of academic burn-out.

**Conclusion:**

Based on the research results, it can be stated that the variables of sleep quality and social intimacy are the predictor factors of academic burn-out.

## 1. Introduction

Academic burnout and academic failure are one of the important concerns of the families and those involved in education. A large number of students in different countries in the world face a phenomenon called academic failure every year. This phenomenon and similar phenomena like academic burnout cause huge economic losses and require the re-taking of the classes or re-spending money on them (Biyabangard, 2001). In this regard, it is worth noting that academic burnout has different components which can be summarized in the three dimensions of the individual, family, school and community, and disorder in any one of them can subject the leaner’s to high risk behaviors (Afshari, 2006).

The term “burnout” was primarily defined Freudenberger (1960) when he observed the signs of fatigue in his employees. He called the first phenomenon “the syndrome of physical-mental force” which is developed in aid practitioners who spend a lot of working hours with other people. Burnout is a syndrome which occurs through mental fatigue and loss of competency and analyzes unfavorable physical conditions and stress professional conditions which probably influence burnout. Weinberg & Creed (2000) believed that burnout is a physical-mental syndrome along with fatigue which leads to negative behaviors and attitudes towards oneself, work and clients, non-productive work and absenteeism from activities, low behavior and dissatisfaction.

Although burnout is considered as a disorder related to the workplace (Maslach, Schaufeli, & Leiter, 2001), this concept can also be useful for conditions related to school and education as well. School is a place where students work; that is to say, they enter the classes and do their homework in order to pass their exams and earn a rank and degree. Therefore, the concept of burnout can extend far beyond the career realm and into the academic realm.

Zhang, Gan, and Cham (2007) realized that academic burnout among the learners refers to the sense of fatigue, cynical and uninterested attitude towards school assignments and sense of competency as learners. Based on the main theory of burnout (Schaufeli et al., 2002), it can be stated that academic burnout includes the three components of exhaustion, cynicism and loss of efficacy. Exhaustion emerges as the sense of pressure specifically chronic fatigue from working too much in school activities. Cynicism and depersonalization emerge as the pessimistic and cynical attitude and indifference to school assignments. Lack of interest in the activities related to education and considering them to be pointless and unimportant. Finally lack of efficacy related to school emerges as states like low sense of competency, low progress and lack of sense of success in school assignments and on the whole, in school. The concept of academic burnout also overlaps with some concepts such as the sleep problem, concern and rumination. However, it should be noted that what distinguishes them is that mental pressure, fatigue, anxiety and symptoms of depression are not related to a special and specific situation but academic burnout is related to a special condition; that is, burnout is measured only in the academic conditions (Ahola & Hakanen, 2007).

Part of the poor academic performance of the learners is attributed to their inadequate sleep. Changes in biological, psychological and social norms that take place in early youth are related to sleep duration, inadequate sleep, irregular sleep schedule and insomnia at this period. Such negative changes in the structure of sleep can have mental-social outcomes such as depressed mood, behavioral problems and also academic problems (Moore et al., 2009). In some studies, researchers managed to actively manipulate sleep and observe behavioral and neuro-cognitive outcomes such as learning, memory capacity and academic performance. These findings strongly showed that: students of different educational levels have chronic lack of sleep or suffer from low sleep quality and frequent daytime sleepiness. Sleep quality and quantity is closely related to learning capacity and academic performance. Lack of continuous sleep is related to declarative and procedural learning in students.

In some studies where sleep was actively limited or optimized, academic and neuro-cognitive performance worsened and improved respectively. Curcio, Ferrara, and De Gennaro (2006) investigated the relationship between sleepiness and sleep deprivation in the learning ability and academic performance of the learners. The results on a large sample of the learners indicated that: sleep quality and quantity is significantly related to the capabilities and performance of the learners. Lack of sleep and sleepiness usually lead in reduction of oral and written performance in the learners. Moore et al. (2009) in a study investigated the relationship between sleepiness, sleep time, variability, sleep time and psychological performances (anxiety, depression) in the youth. The results indicated that sleepiness has led to high scores on the anxiety and depression scales (Ehteshamzade & Mar’ashi, 2009).

One of the other factors that affects academic burnout of the learners is the level of their social intimacy with people in the society; that is, creation of intimacy or the capacity for openness of and participation in bilateral relationships with other people. The ability to establish intimate relations with others is considered as one of the key factors in mental health and well-being of people (Montgomery, 2005). In order to get prepared for this potential advantage, young people go through the following steps: the person’s initial relations with the caregivers (in childhood), the starting of initial relations with the peers (in adolescence) and finally, entry into adulthood when at best, people develop the capability of establishing long-term intimate relations that bring mutual trust. Such relations in turn support and enhance personal health and well-being (Daneshvarpoor et al., 2007).

The social intimacy network affects stress, specifically occupational and academic stress. Such intimacies in academic environments lead to the increase of a person’s ability to deal with stress (Bradley & Cratwright, 2002) and increase of efficacy in educational environments and increase of satisfaction and reduction of mental weariness and burnout (Hall, 2007). Strazdins and Broom (2008) believe that social relations and consequently social intimacy can almost in every way act as a shield against pressing incidents and events and protect a person from adverse consequences and its lack can be stressful for the person and bring about some problems as well. Brouwer et al., (2008) in their study showed that there is an inverse relationship between the scores of the social intimacy and support scale and the level of depression of university students. As the sleep quality of the learners and the level of their social intimacy play a significant role in protection of health well-being and will directly affect the reduction of stress and burnout in academic conditions, the present study has investigated the relationship between sleep quality and social intimacy, and academic burnout.

## 2. Materials and Methods

The present study was descriptive and correlational. Its objective was to investigate the relationship between sleep quality and social intimacy, and academic burnout. The population of the present study were medical students of Tabriz University of Medical Sciences who were selected in the proportional stratified random sampling method. They were 196 female and male students.

### 2.1 Research Tools

**Miller Social Intimacy Scale (MSIS):** This scale, with 17 items (items 4 to 20) was designed by Miller and Lefcourt (1982) for assessment of the level of intimacy and closeness of a person with other persons. It is a 5-point scale (A=1, B=2, C=3, D=4, E=5). The items 1, 2, 3, 21 and 22 are not scored. The items 5 and 17 have inverse scores; then, each of the items is added to obtain the total score. Higher scores will indicate higher intimacy. The reliability for the social intimacy scale was obtained 85% through Cronbach’s alpha

**Pittsburgh Sleep Quality Index (PSQI):** This questionnaire was made with the goal of investigation of sleep quality over the last month and is comprised of 18 items. It has been stated that this questionnaire can distinguish the bad and good sleep qualities (0.36). Each of the seven scales (1. Subjective sleep quality, 2. Delay in falling sleep, 3. Sleep duration, 4. Useful sleep, 5. Sleep disorders, 6. Taking sedatives, 7. Daily dysfunction) has had internal validity and reliability coefficient of about 83% and 36% respectively. The reliability of these tools in the present study was estimated as 64%. Each of these sub-scales are allocated scores 0 to 3, according to which: no times=0, less than once a week=1, once or twice a week=2, and three times or more=3. The total score of the questionnaire ranges between 0 and 21. High scores indicate poor sleep quality. The total score larger than 5 shows that a person with poor sleep has severe problems at least in two areas or average problems in more than three areas.

**Berso et al. Academic Burnout Questionnaire:** This questionnaire has been designed by Berso et al. (1997). This questionnaire assesses the three areas of academic burnout, which are academic fatigue, lack of academic interest and academic failure. The aforementioned questionnaire has 15 items and has been graded from “strongly disagree” to “strongly agree” using Likert’s 5-point scale. Academic fatigue (the lessons are boring) has 5 items, lack of academic interest (I feel I have no interest in the lessons) has 4 items and academic failure (I feel I can’t cope with academic problems) has 6 items. The makers have calculated the questionnaire reliability as 70%, 82% and 75% for the three dimensions of academic burnout respectively. In the present study, the reliability of the questionnaire has been obtained as 77% (Neami, 2009). The questionnaires was delivered in paper form and submitted anonymously. Participation was voluntary and submitted of a completed questionnaires were considered as consent.

## 3. Results

**Research question 1:** Is there a relationship between sleep quality and academic burnout of students?

The findings of Table 1 show that there is a significant and positive difference between the two variables of sleep quality and academic burnout at the level 0.001. Based on this finding, it can be concluded that the more sleep quality people have, the better they will manage to make progress in terms of educational opportunities and the less will they suffer from academic burnout. As the study by Eliasson et al. (2002) showed a part of the poor academic performance of the students is attributed to their inadequate sleep. In some studies, researchers managed to manipulate sleep actively and observe behavioral and neuro-cognitive outcomes like learning, memory capacity and academic performance. These findings strongly show that sleep quality and quantity is closely related to learning capacity and academic performance (Curcio, et al., 2006).

**Table 1:** The results of correlation coefficient between sleep quality and academic burnout of the students

Variable	Statistic (correlation coefficient)	Significance level
Sleep quality and academic burnout	0.387	0.001

**Research question 2:** Is there a relationship between social intimacy and academic burnout of students?

The findings of Table 2 show that there is a significant and inverse difference between the two variables of social intimacy and academic burnout at the level 0.001; that is to say, social intimacy can be a good predictor for academic burnout and the more social intimacy people have and the more they can get the support of a specific person and have warm and close and committed cooperation, the less will they suffer from academic burnout.

**Table 2:** The results of correlation coefficient between social intimacy and academic burnout

variable	Statistic (correlation coefficient)	Significance level
Social intimacy and academic burnout	**0.405-**	0.001

It can also be observed in Table 3 that there is an inverse and negative relationship between all the components of academic burnout and social intimacy. The people that have high social intimacy will suffer less from failure and lack of interest and academic fatigue.

**Table 3:** The results of correlation coefficient between social intimacy and the components of academic burnout

	Variables	Number	Correlation coefficient	Significance level
Academic burnout	Failure	196	37.0-	0.001
	Lack of interest	196	29.0-	0.001
	Academic fatigue	196	38.0-	0.001

**Table 4:** Summary of multiple regression analysis model of predictor factors of academic burnout

Model	R	R2	Adjusted R2	SE	Durbin-Watson statistic of model 2
1	0.38	0.15	0.14	9.93	1.68
2	0.55	0.3	0.29	8.99	

**Research question 3:** Can the variables of sleep quality and social intimacy predict academic burnout?

As can be seen in the above table, in the first step, when the variable of sleep quality entered the equation, its squared correlation was (0.15); that is, it explained 14% of the variance of academic burnout and after the entry of the second variable, which is social intimacy, the level of (variance) explanation increased to 30% and therefore here the variable of social intimacy explained only 15% of the variance of academic burnout. Thus, it can be said that on the whole 30% of the variance in academic burnout is related to the variance of “sleep quality” and “social intimacy” variables. Based on these results, the second model was selected as the final model based on higher explanation power (R^2^=0.30).

As Table 5 shows, both variables entered in the regression model significantly predict the criterion variable (p ≥0.05 and F_a and 193_= 34.17).

**Table 5:** ANOVA test for the significance of model 2 of regression

Source of variance	Sum of squares	Degrees of freedom	Mean square	F	Significance level
**Regression**	6888.48	2	**34.177**	34.177	0.001
**Remaining**	15618.515	193			
**Total**	22507	195			

**Research question 4:** Do the students with the sleep quality and social intimacy profiles differ in terms of academic burnout?

Study of F-statistic in the Shapiro–Wilk’s test in (MANOVA) on different groups during the implementation of cluster analysis showed that classification of students into two groups has the highest significant value; therefore, the second group state was used for grouping or clustering. In this study, the clustering criteria were their sleep quality (subjective sleep quality, delay in falling asleep, useful sleep, sleep disorders, taking sedatives and daily dysfunction) and social intimacy. As cluster analysis is sensitive to outlier data, this assumption was investigated in the research question. Also, Euclidean square was used as an index of similarity. The number of the clusters was determined through multivariate analysis of variance (MANOVA).

As can be seen in Table 6, the mean and standard deviation of each of the research variables have been shown in clusters 1 and 2. Based on the students’ scores in the research variables each cluster is give none title. Cluster 1 includes students who have a high level of mean in the variables of social intimacy and have a low level of mean in the variables of subjective sleep quality, delay in falling asleep, sleep duration, taking sedatives and daily dysfunction. Accordingly, cluster 1 was named as “people with high social intimacy”. In cluster 2, people in the variable of social intimacy have a low level of mean and have a high level of mean in the variables of taking sedatives, daily dysfunction, subjective sleep quality, sleep duration and delay in falling asleep. Based on these means, cluster 2 was named as “people with low social intimacy”.

**Table 6:** The mean and standard deviation of the research variable scores based on the clusters

Research variables	Cluster 1		Cluster 2	
	
	Mean	Standard deviation	Mean	Standard deviation
Social intimacy	**70.77**	6.58	29.49	7.93
Subjective sleep quality	**0.94**	0.77	1.08	0.11
Delay in falling asleep	**1.5**	1.81	1.98	1.81
Sleep duration	**6.56**	1.44	6.8	1.3
Useful sleep	**0.0**	**0.0**	**0.0**	**0.0**
Sleep disorders	**1.05**	0.68	1.06	0.79
Taking sedatives	**0.54**	0.87	0.59	0.78
Daily dysfunction	**1.07**	0.89	1.13	0.71

In order to determine the students’ profiles in terms of academic burnout, the independent t-test has been used.

Based on Table 7, it can be observed that this research question got the required support; that is to say, there is a significant difference between cluster 1 and cluster 2 in the variable of academic burnout (p>0.001). The given difference results from the different and real performance of people in clusters 1 and 2.

**Table 7:** The t-test results for investigation of the performance difference of cluster 1 & 2 on the variable of academic burnout

	Group	Number	Mean	df	t	Significance level
Academic achievement	Cluster 1	**135**	33.70	**123**	**96.-4**	0.001
	Cluster 2	**61**	41.31			

## 4. Discussion

Burnout is a state of mental and emotional fatigue which is a result of chronic stress syndrome including role overload, pressure and time constraint and lack of necessary resources to carry out the assigned school tasks and assignments. They usually experience symptoms such as lack of interest in the lessons, inability to maintain a continuous presence in the classroom, lack of participation in class activities, sense of meaninglessness in class activities and sense of inability in learning the lessons. In this study, the relationship between sleep quality and social intimacy, and academic burnout has been investigated. The first research question indicates a significant relationship between sleep quality and academic burnout; that is to say, the higher sleep quality people have, the less will they have performance failure and the less will they suffer from academic burnout. The results of the study by Quraishi and Aghajani (2008) showed that sleep quality was higher in people with the average above 16. This relationship can show the improvement of academic performance with the increase of sleep quality.

The results are aligned with the results of the studies by Moore et al. (2009), Curcio et al. (2006), Rosen et al. (2004), and Mayers et al. (2003). Accordingly, it can be said that increase of sleep quality will lead to the reduction of sleepiness in the students and consequently improvement of their overall performance.

The second research question shows that there is a significant and inverse relationship between social intimacy and academic burnout. People that have high social intimacy and get the support of the friends or the family less frequently suffer from burnout with regard to school. As the third research question showed, social intimacy alone was able to predict 15% of the variance of academic burnout. The results of these questions are in line with the studies by Zhang et al. (2007), Bradley and Cartwright (2002), Hall (2007) and Strazdins et al. (2008).

On the other hand, it was observed in the present study that the students were considered in two separate clusters in terms of the variables of sleep quality and social intimacy. Cluster 1 consisted of people that had high social intimacy and low sleep disorder; cluster 2 was comprised of people that had low social intimacy and high sleep disorder and in fact these two clusters had a significant difference in terms of academic burnout.

## 5. Conclusion

Burnout leads to mental distresses such as anxiety, depression, repression, hostility or fear. These people usually experience symptoms like lack of interest in the lessons, inability to maintain a continuous presence in the classroom, lack of participation in class activities, sense of meaninglessness in class activities and sense of inability in learning the lessons. Therefore, based on the research results, both variables of social intimacy and sleep quality were recognized as the factors effective in burnout so that if these two factors be high, people will suffer less from academic burnout.
